# Quantitatively measured tremor in hand-arm vibration-exposed workers

**DOI:** 10.1007/s00420-014-0959-0

**Published:** 2014-07-05

**Authors:** Maria Edlund, Lage Burström, Mats Hagberg, Ronnie Lundström, Tohr Nilsson, Helena Sandén, Gunilla Wastensson

**Affiliations:** 1 Department of Occupational and Environmental Medicine, Sahlgrenska School of Public Health and Community Medicine, University of Göteborg, Göteborg, Sweden; 2 Occupational and Environmental Medicine, Department of Public Health and Clinical Medicine, Umeå University, Umeå, Sweden; 3 Department of Occupational and Environmental Medicine, Sundsvall Hospital, Sundsvall, Sweden

**Keywords:** Tremor, Hand-arm vibration exposure, Hand-arm vibration syndrome, CATSYS Tremor Pen^®^, Neurological

## Abstract

**Objectives:**

The aim of the present study was to investigate the possible increase in hand tremor in relation to hand-arm vibration (HAV) exposure in a cohort of exposed and unexposed workers.

**Methods:**

Participants were 178 male workers with or without exposure to HAV. The study is cross-sectional regarding the outcome of tremor and has a longitudinal design with respect to exposure. The dose of HAV exposure was collected via questionnaires and measurements at several follow-ups. The CATSYS Tremor Pen^®^ was used for measuring postural tremor. Multiple linear regression methods were used to analyze associations between different tremor variables and HAV exposure, along with predictor variables with biological relevance.

**Results:**

There were no statistically significant associations between the different tremor variables and cumulative HAV or current exposure. Age was a statistically significant predictor of variation in tremor outcomes for three of the four tremor variables, whereas nicotine use was a statistically significant predictor of either left or right hand or both hands for all four tremor variables.

**Conclusions:**

In the present study, there was no evidence of an exposure–response association between HAV exposure and measured postural tremor. Increase in age and nicotine use appeared to be the strongest predictors of tremor.

## Introduction

The symptoms that compose the hand-arm vibration syndrome (HAVS) have previously been extensively described and are referred to as mainly vascular, neurological and muscular (Chetter et al. 1998; Heaver et al. 2011). The most prominent symptoms are made up of vascular and peripheral neurological disorders (i.e., sensorineural), where the latter symptoms are described as the most frequent and also the most resistant to recovery (Chetter et al. 1998; Futatsuka et al. 1989; Koskimies et al. 1992). The HAVS is a complex condition, and it has been suggested that all involved signs and symptoms are not yet discovered (Griffin 2008). Several symptoms associated with or possibly associated with the syndrome have been explored in previous studies, and as early as the beginning of the twentieth century, the symptom of tremor was mentioned among vibration-exposed workers (Bylund et al. 2002; Futatsuka et al. 2005; Griffin 1997).

However, the studies investigating tremor among HAV-exposed workers are few, and one of the studies was conducted on only women (Bylund et al. 2002; Futatsuka et al. 2005). Thus, little is known about tremor as a symptom possibly associated with prolonged HAV, and to our knowledge, there has been no previous study on quantitative measurements of tremor in HAV-exposed workers. According to Deuschl et al., peripheral mechanisms may cause some types of tremor (Deuschl et al. 1996). It has been observed that patients with acquired and hereditary peripheral neuropathies exhibit differing forms of tremor and more often than compared to a control group (Elble 2009; Wasielewska et al. 2013), but no exact pathophysiological pathways have been revealed (Elble 2009). The various neurological disorders in the HAVS are not clearly defined, and their form is poorly understood (Griffin 2008). Neurological symptoms including tremor can be disturbing and also potentially disabling. In view of these facts, and also because of clinical observations of tremor in HAV-exposed patients, further exploration is desirable.

### Aim

The aim of the present study was therefore to investigate the possible increase in hand tremor in relation to prolonged HAV exposure in a cohort of exposed and unexposed workers.

## Methods

### Study design

The study design was cross-sectional with regard to the outcome of tremor measurements and longitudinal for exposure assessment (except for a minor part of workers (*n* = 34), who extended the cohort in 2008). There was an upper age limit of 55 years.

### Participants

The majority of the participants were originally recruited in 1987 and 1992, from an engineering plant in Sweden, to create a cohort baseline in 1992, with follow-ups conducted in 1997, 2002 and 2008, concerning a HAV exposure assessment.

In 1992, the cohort comprised 241 male participants, 181 of whom were exposed to HAV while the remaining participants were unexposed. All exposed workers were invited to constitute the cohort, and approximately all of them accepted participation. The unexposed workers were selected from a larger group of approximately 500 employees at the engineering plant, and they were therefore randomly selected and invited from the payroll roster, resulting in *n* = 60 unexposed workers in 1992 (Edlund et al. 2013).

In 2008, all new manual (HAV exposed) employees (*n* = 34) were invited to extend the cohort, resulting in 275 invited subjects, 189 of whom eventually performed the tests. The main reasons for not participating were retirement and moving to another area. Due to exclusion criteria, there were eventually 178 participants remaining for the statistical analyses.

Exclusion criteria were diabetes, information on alcohol abuse and neurological diseases such as multiple sclerosis, stroke and/or polyneuropathy.

### Clinical examination, interview and questionnaires

An occupational medicine physician (T.N.) conducted the clinical examinations and performed the interviews on all participating workers. The physical examination concerned principally the hands and upper limbs with a focus on neurological and musculoskeletal disorders. A standard procedure was followed, including a basic neurological examination of the hands.

In the medical interview, the participants were asked to give a detailed history of previous and current diseases and symptoms, medication, alcohol consumption, and nicotine use. Nicotine use concerned either smoking or snuff use. A supplementary questionnaire was also administered covering the above-mentioned information.

The different examinations and tests were all conducted in 1 day starting with the medical examination, followed by (in given order) testing of manual dexterity; finger and hand strength; touch, vibrotactile, and thermotactile sense; and the quantitative tremor measurements.

### Vibration exposure assessment

Information on HAV exposure was collected via a questionnaire and measurements (Edlund et al. 2013). The workers noted debut of exposure (age or year), exposure in minutes per day, and type of tool and work task. Leisure time exposure was included in the total exposure dose. Measurements were conducted according to a standardized scheme during representative working cycles. The main tools used by the participating workers were grinders, die grinders and hammers with vibration intensity ranging from 1.5 to 10 m/s^2^.

HAV exposure was given in time (hours) and acceleration level (m/s^2^) in accordance with International Organization for Standardization (ISO) guidelines (European Council; ISO:5349-1; ISO:5349-2). The product of exposure hours (h) and of hand-arm acceleration (m/s^2^) was used as the cumulative HAV exposure dose (unit h m/s^2^). As an example, a worker who operates a hand-held vibrating tool with the intensity of 2.5 m/s^2^ (the EU action level) during 8 h per working day and 220 working days per year for 1 year ends up with an exposure dose of 4,400 h m/s^2^. The cumulative dose of HAV in 2008 was calculated from measurements and questionnaires in 1987, 1992, 1997, 2002 (only questionnaire) and 2008.

Current exposure, as in using hand-held vibrating tools at the time of follow-up (2008), was recorded in acceleration (m/s^2^) and given in A(8) values (ISO:5349-1) that ranged from 0.0 to 2.1 m/s^2^ with a mean of 0.50 m/s^2^ and standard deviation (SD) of 0.80 m/s^2^.

### Quantitative tremor measurements

The subjects were asked (in advance) to refrain from HAV exposure and nicotine use, on the day of testing. The measurements were conducted by an experienced physiotherapist.

The CATSYS Tremor Pen^®^ was used for measuring postural tremor (DPD 2000). The equipment consists of a biaxial micro-accelerometer embedded in a low-mass stylus (12 cm × 0.8 cm), which is sensitive when perpendicular to the central axis of the stylus, and has been standardized and validated (Despres et al. 2000; Edwards and Beuter 1997). For the testing procedure, the participants were asked to sit in a chair and hold the stylus as they would hold a writing pen, with the elbow joint bent at an angle of 90°, and to avoid contact. The stylus was held horizontally about 10 cm in front of the navel. Tremor was recorded successively in each hand over 16.4 s. The participant was asked to look at the tip of the stylus and breathe normally during recording.

The tremor registrations were displayed in real time on a time axis plot on the computer screen. Fourier transformation was used to determine the power distribution across a frequency band varying from 0.9 to 15 Hz. Four different measures calculated by the CATSYS software were used: tremor intensity, center frequency, frequency dispersion and harmonic index (Table 1).

**Table 1:** Definitions of measures used to characterize postural arm tremor recorded with the CATSYS system (Despres et al. 2000; Wastensson et al. 2006)

Characteristics^a^	Definitions
Tremor intensity, (m/s^2^)	The tremor amplitude given in root-mean-square of acceleration (m/s^2^) recorded in the 0.9- to 15-Hz band. Higher values indicate more tremor
Center frequency (CF), (Hz)	The median frequency of the acceleration in the 0.9- to 15-Hz band. Abnormal scores are expected to be lower
Frequency dispersion (FD), (Hz)	The standard deviation of CF indicating the degree of tremor irregularity. Regular tremor has low values of FD. Abnormal scores are expected to be lower
Harmonic index (HI)	Comparison of the tremor frequency pattern with a single harmonic oscillation. The HI decreases when the tremor is composed of many oscillations. Abnormal scores are expected to be higher

^a^Definitions of characteristics from Danish Product Development Ltd. (DPD 2000)

### Statistical analyses

Descriptive statistics are given in means, SDs or percentages. Data on the different tremor variables are given in means and SD. Student’s *t* test for comparison of independent groups (unexposed/exposed workers) was used for age, BMI and alcohol consumption.

Multiple linear regression analyses were conducted to assess the associations between the tremor variables as outcomes (dependent variables) and HAV exposure. The backward elimination and forward selection methods were used. Predictor or explanatory variables of biological relevance (age, alcohol consumption, nicotine use, current exposure) were entered in the model.

Analyses were conducted with the assumption of normal distribution, and the *p* values <0.05 level was considered statistically significant.

Statistical analyses were performed using PASW Statistics 18.0 (SPSS Inc., Chicago, IL, USA).

### Ethical approval

Informed consent was obtained from each participant. The Regional Ethics Committee of Umea University approved the study, which was performed in accordance with the ethical standards detailed in the 1964 Declaration of Helsinki and its later amendments.

## Results

### Descriptive data

Table 2 presents the characteristics of the study population. The unexposed workers were older than the exposed workers, but did not differ concerning BMI, alcohol use, medication or diabetes. Nicotine use was more common among the exposed workers (Table 3).

**Table 2:** Characteristics of study population

Variable	Unexposed (*n* = 39)			Exposed (*n* = 139)		
	Mean	SD	%	Mean	SD	%
Age (years)	58	10		53	11	
Body mass index	26	4		27	4	
Alcohol (cl/week)	21	14		23	21	
Nicotine use (%)			15			41
Thyroid disease (%)			4.8			1
Diabetes (%)			2.3			2
Self-reported use of medication (Beta-2-agonists/antagonists) (%)			11			11
Cumulative HAV exposure (h m/s^2^)				31,600	27,700	
Cumulative HAV exposure (days)				615	450	

*HAV*  Hand-arm vibration,* h*  hours, *day*  working day of 8 h

**Table 3:** Data on tremor measurement values using the CATSYS system

	Unexposed (*n* = 39)		Exposed (*n* = 139)	
	Mean	SD	Mean	SD
Tremor intensity (m/s^2^), R	0.129	0.058	0.138	0.060
Tremor intensity (m/s^2^), L	0.122	0.045	0.122	0.049
Center frequency (Hz), R	7.22	1.04	7.35	0.906
Center frequency (Hz), L	7.11	1.38	7.38	1.12
Frequency dispersion (Hz), R	2.89	0.681	2.70	0.657
Frequency dispersion (Hz), L	3.08	0.754	3.17	0.696
Harmonic index, R	0.914	0.033	0.920	0.029
Harmonic index, L	0.898	0.040	0.892	0.419

*SD* standard deviation, *R* right hand, *L* left hand

### Tremor measurements

Multiple linear regression models with the different tremor variables as outcome yielded associations with either age or nicotine use, or both, but no association with either cumulative HAV exposure or current exposure. (If using two separate models for cumulative and current HAV exposure, the results were the same.)

Age resulted in a statistically significant predictor for more pathological values concerning tremor intensity (left hand), in other words higher values; frequency dispersion (both hands), in other words lower values; and harmonic index (both hands), in other words higher values. Nicotine use was also presented as statistically significant for more pathological values of tremor for both hands concerning tremor intensity (i.e., higher values), and concerning frequency dispersion (i.e., lower values). For the left hand, there were more pathological values for harmonic index (i.e., higher values). Center frequency showed an association for less pathological tremor values for the right hand (i.e., higher values). Table 4 presents adjusted *R* ^2^ values, regression coefficients, *p* values of *F* tests and statistically significant predictors (age and nicotine use).

**Table 4:** Results from the multiple linear regression models with the different tremor variables as outcomes, including age and nicotine use as predictors, *p* values of adj *R* ^2^ and *F* test, and regression coefficients

	adj *R* ^2^, *p* value	*F* test, *p* value	Age, *p* value	Age, regression coefficient	Nicotine use, *p* value	Nicotine use, regression coefficient
Tremor intensity (m/s^2^), R	0.0785	0.0004	ns		0.0001	0.0368
Tremor intensity (m/s^2^), L	0.111	<0.0001	0.0014	0.001	<0.0001	0.0320
Center frequency (Hz), R	0.0394	0.0122	ns		0.0494	0.287
Center frequency (Hz), L	0.00264	0.296	ns		ns	
Frequency dispersion (Hz), R	0.0473	0.0060	0.0370	−0.0099	0.0037	−0.305
Frequency dispersion (Hz), L	0.0339	0.0198	0.0146	−0.013	0.0478	−0.224
Harmonic index, R	0.0257	0.0403	0.0292	0.00048	ns	
Harmonic index, L	0.0955	<0.0001	<0.0001	0.00127	0.0420	0.0130

*R* right, *L* left, *Hz* hertz, *adj* adjusted, *ns* not statistically significant

In general, the adjusted *R* ^2^ values were very low and the model with center frequency for the left hand did not hold (the *p* value for *F* test was above the 0.05 level).

## Discussion

There were no statistically significant associations between the different tremor variables and cumulative HAV or current exposure. Age was a statistically significant predictor of variation in tremor outcome for three of the four tremor variables, whereas nicotine use was a statistically significant predictor of either left or right hand or both hands for all four tremor variables. Measured values were in accordance with values normally occurring in a healthy population (Despres et al. 2000).

The previous reports on tremor occurrence mentioned in the introduction (Bylund et al. 2002; Futatsuka et al. 2005) may possibly be explained by different interpretations of the definition of tremor. There are no clear definitions of tremor in the studies reporting tremor in HAV-exposed workers (Bylund et al. 2002; Futatsuka et al. 2005). Futatsuka et al. seem to have used interviews and Bylund et al. used a questionnaire based on “earlier surveys” from, for instance, Atroshi et al. (Atroshi et al. 1998). Shivers, jerks and possibly impaired manual dexterity may be mistaken for or perceived as tremor. According to Sakakibara et al., loss of sensory function and/or muscular dysfunction in the hands and fingers may be associated with impaired manual dexterity, which could possibly explain symptoms that subjects describe as similar to tremor (Sakakibara et al. 2005). One possible mechanism for impaired manual dexterity could be temporary numbness due to acute effects of HAV exposure (Griffin 2008). Furthermore, tremor may have many causal explanations and is a common symptom in the general population, which may also be reflected in the working population exposed to HAV (Deuschl et al. 1996). Obviously, it may be difficult to distinguish tremor from other symptoms as well as classify type of tremor (Alty and Kempster 2011). Consequently, this should give more credibility/strength to the present study with quantitatively measured tremor.

Increased tremor, usually postural, has been reported among patients with neuropathies of different origin (Elble 2009; Wasielewska et al. 2013); however, there is a possibility that the degree of nerve affection among the workers in the present study population is not severe enough to cause tremor.

Tremor has been hypothesized to depend on acute effects of HAV exposure; however, one study with an experimental approach testing acute effects after a limited dose of HAVs showed the opposite, in other words, less tremor after exposure (Gomez et al. 2003). Precautions were taken in the present study trying to avoid acute effects from HAVs, and as far as we know, the participants were not exposed on the day of tremor measuring.

Nicotine use and age have to be accounted for when comparing groups with respect to tremor. Increase in age is known to affect tremor, and it has been shown that tremor frequency decreases with age (Despres et al. 2000). The present study resulted in more pathological tremor values with increasing age. It has been suggested that age-related changes in tremor could be explained by a degradation of the motor control (Almeida et al. 2010). As for nicotine users, there is prior knowledge that nicotine users have higher tremor intensity than non-nicotine users and that older age may be a predictor of importance for the quantity of tremor in nicotine users, in contrast to non-nicotine users (Ellingsen et al. 2006). Furthermore, nicotine users have exhibited lower frequency dispersion compared to non-nicotine users (Ellingsen et al. 2006). Thus, the results of nicotine use in the present study are in accordance with previous findings.

Information on HAV exposure has been collected prospectively both from questionnaires and via measurements for a clear majority of the participants. This procedure of careful collection and assessment of data gives strength to the study and minimizes the possibility of information bias and misclassification of workers in the different quartiles. Furthermore, a study comparing a neurologist’s physical examination to quantitative measurements of tremor disclosed that the latter method provided more precise results (Gerr et al. 2000). All tremor measurements concern postural tremor, and it cannot be entirely ruled out that effects from HAV exposure could have an impact on some other form of tremor such as, for instance, kinetic tremor or task-specific tremor.

## Conclusion

In the present study, there was no evidence of an exposure–response association between HAV exposure and measured postural tremor. Increase in age and nicotine use appeared to be the strongest predictors of tremor.
